# An Optimal Current Observer for Predictive Current Controlled Buck DC-DC Converters

**DOI:** 10.3390/s140508851

**Published:** 2014-05-19

**Authors:** Run Min, Chen Chen, Xiaodong Zhang, Xuecheng Zou, Qiaoling Tong, Qiao Zhang

**Affiliations:** 1 School of Optical and Electronic Information, Huazhong University of Science and Technology, Wuhan 430074, China; E-Mails: hustminrun@gmail.com (R.M.); chenchen_hust@163.com (C.C.); sanctum.zhang@gmail.com (X.Z.); tongqiaoling@hust.edu.cn (Q.T.); 2 School of Automation, Huazhong University of Science and Technology, Wuhan 430074, China; E-Mail: zhangqiao@263.net

**Keywords:** converter, DC-DC, buck, PCC, observer

## Abstract

In digital current mode controlled DC-DC converters, conventional current sensors might not provide isolation at a minimized price, power loss and size. Therefore, a current observer which can be realized based on the digital circuit itself, is a possible substitute. However, the observed current may diverge due to the parasitic resistors and the forward conduction voltage of the diode. Moreover, the divergence of the observed current will cause steady state errors in the output voltage. In this paper, an optimal current observer is proposed. It achieves the highest observation accuracy by compensating for all the known parasitic parameters. By employing the optimal current observer-based predictive current controller, a buck converter is implemented. The converter has a convergently and accurately observed inductor current, and shows preferable transient response than the conventional voltage mode controlled converter. Besides, costs, power loss and size are minimized since the strategy requires no additional hardware for current sensing. The effectiveness of the proposed optimal current observer is demonstrated experimentally.

## 1. Introduction

In recent years, predictive current control (PCC) was found to be a robust, fast and easy control strategy for digitally controlled DC-DC converters in continuous current mode (CCM). Therefore, it has been extensively studied by many researchers [1–4]. Bibian *et al.* investigated a high performance PCC based on dead-beat control strategy [3], which shows a great advantage of low calculation complexity. However, the response is quite slow due to its current error elimination once every four switching cycles. A fast response PCC strategy is proposed by Chen *et al.* [4], whereby the disturbance of the inductance current can be eliminated in two switching cycles, whether in the valley, peak or average current control modes. Many PCC strategies have been proposed recently, but all the strategies need to sample the inductor current using a current sensor.

Different current sensing techniques meet different applications in terms of cost, size, accuracy, isolation, *etc.* A thorough review of state-of-the-art current sensing techniques is presented in [5]. Sampling by a shunt resistor is found to be very simple, but is non-isolated and this causes high power losses. By using a complementary matching filter, sampling by the intrinsic resistance of the inductor brings no additional power losses and is very low-cost. However, the thermal drift is distinct and the accuracy could be low due to the mismatches between the filter and the power inductor [6,7]. Another non-isolated current sensing technique uses the mirroring circuit, which is widely employed in integrated circuits. However, this method may suffer from EMI problems and the accuracy might be low due to the amplifier offset and the mismatch between the power Field Effect Transistor (powerFET) and the sense Field Effect Transistor (senseFET) [8,9]. In order to improve the EMI immunity, a new mirroring circuit was proposed, which exploits the Miller effect on the switching transient of the powerFET [10]. All the non-isolated current sensors are electrically connected to the power converter, thus lowering the safety level. For isolation, other current sensors have to be used, including Hall effect sensors, fluxgate sensors, Rogowski coil sensors, anisotropic magnetoresistance (AMR) effect sensors, the giant magnetoimpedance (GMI) sensing system, *etc.* Hall-effect sensors are non-contacting to the power stage and have reasonable accuracy, bandwidth and power loss [11], however, they are expensive, and may suffer EMI problems if the signal amplification chain is not properly designed [12]. For the EMI issue, a new contactless current sensor based on the Hall effect is proposed to increase the immunity to the radio frequency interference collected by the cables [13]. Fluxgate sensors have the highest performance at the cost of the highest price [5]. Rogowski coil sensors provide a wide measuring range and a high accuracy at relatively low cost [5]. Closed-loop AMR-based sensors have better performance than Hall-effect sensors, but smaller measuring range and higher cost [5]. The GMI sensing system provides isolation with very low-cost [14,15]. Nevertheless, the system requires frequent calibration due to its sensitivity to time and temperature.

The current sensors mentioned above all have different advantages and drawbacks, and thus could meet different requirements. However, existing techniques might not suit applications which require isolation with minimal price, power loss and size. Therefore, a current observer (CO) turns out to be a suitable substitute for conventional current sensors in digitally controlled converters. The cost, size and power consumption can be reduced since it does not need any auxiliary hardware, even though the accuracy might be affected by the voltage ripple or the mismatches between the observer and the converter.

Current observers are used for motor controls, fault detection, and were firstly introduced for DC-DC applications by Midya [16]. The paper proposed an analog observation method for sensorless analog current mode control. However, the implementation does not suit digital controllers. For digitally controlled converters, an easy feed forward CO was proposed in 2004 [17]. It can effectively avoid the impact from the output voltage on the observed current. This algorithm, however, does not consider the influence of parasitic parameters, and thus has low accuracy. To improve the accuracy, both the equivalent series resistance of the capacitor and the internal resistance of the inductor are considered in the observer, which is used for model predictive control (MPC) [18,19]. The observer can be realized using either a hybrid Kalman filter or an extended Kalman filter (EKF). The two approaches have different complexity, accuracy and linearity. Qiu *et al.* proposed an average current observer for the PCC controller [20], which is relatively accurate due to its consideration of five parasitic parameters, but the compensated current slopes are still not accurate enough. A sensorless adaptive voltage positioning (SLAVP) control scheme was proposed in [21]. The strategy suits applications which require reduced dynamic output-voltage deviation. None of the works mentioned above have studied the convergence of the observed current, nor the steady state errors of the output voltage.

For PCC controllers with the basic current observer, the observed current will diverge due to the forward voltage of the diode and the parasitic resistors in the converter. The divergence of the observed current degrades the reliability of the converter, and will cause calculation result overflow in the digital circuit. More importantly, the divergence further induces steady state errors in the output voltage. In our previously published paper, these issues were discussed in detail, and a compensation strategy based on a boost converter was proposed [22]. However, the compensation strategy cannot be applied to the buck converter because of the different topologies. The difference leads to the need for different mathematical modeling and controller optimization methods. In this paper, an optimal current observer (OCO) is proposed for the buck converter which achieves the highest observation accuracy. By employing compensations for both the observed current and the sampled voltage, the OCO-based PCC controller converges the observed current to the valley value of the inductor current, and eliminates the steady state error of the output voltage. Nevertheless, the observation accuracy could still be affected by a wrongly measured inductance and parasitic parameters.

The paper is organized as follows: Section 2 introduces the basic CO-based PCC algorithm, which includes the observer algorithm and the PCC strategy. In Section 3, the relationship between the convergence of the observed current and the parasitic parameters is derived, and the steady state error of the output voltage is analyzed. In Section 4, the OCO strategy is proposed, which can converge the observed current and eliminate the steady state error by compensating the observed current and the sampled voltage. Experimental results are shown in Section 5 to support the proposed theory, and to prove the effectiveness of the OCO based PCC strategy. Finally, a brief conclusion is given in Section 6.

## 2. Current Observer Based Predictive Current Control

**Figure 1.**
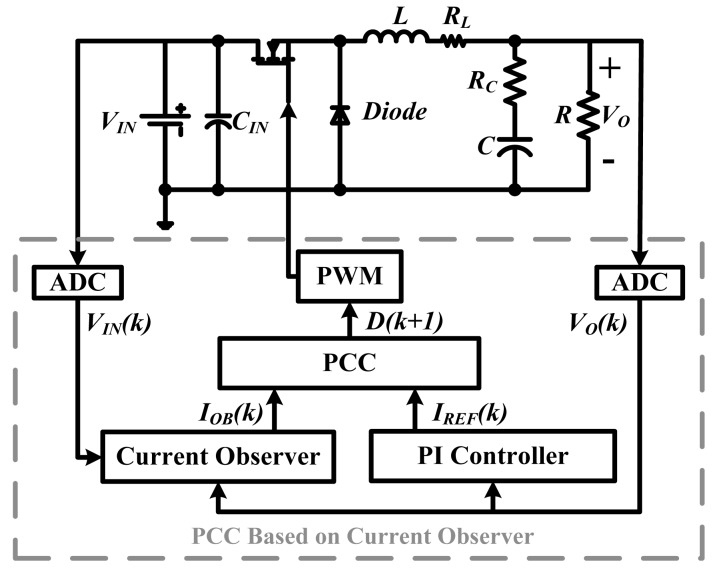
Buck DC-DC converter with the CO based PCC controller.

The construction of buck DC-DC converter with the CO based PCC controller, is shown in Figure 1. The controller is a dual-loop system. The voltage loop is a PI compensator, which outputs the reference current *I_REF_*. The current loop is the PCC controller, which calculates the duty ratio for the next switching cycle. In every switching cycle, the voltage sampling, the PI regulation, the current sensing and the PCC regulation are processed in sequence. In this way, the current error could be eliminated in two switching cycles.

Supposed that the converter works in CCM. Ignoring the parasitic parameters, a current differential equation can be derived based on the average voltage on the inductor, shown as Equation (1):

(1) $$L\frac{dI_{L}(t)}{dt}=DV_{IN}(t)-V_{O}(t)$$

where *I_L_, V_O_, D, R* and *V_IN_* denote the inductor current, the output voltage, the duty ratio, the equivalent load resistor and the input voltage, respectively. The equation is the theoretical base for current observer.

### 2.1. Basic Current Observer

Based on Equation (1), the inductor current can be observed using *D, V_O_, V_IN_, L* and *T*. In detail, for the *k*th switch cycle, the voltage absolute value on the inductor is *V_IN_*(*k*)− *V_O_*(*k*) when the MOS switch is on, while being *V_O_*(*k*) when it is off. So, *M*_1_(*k*) and *M*_2_(*k*) can be written as Equation (2):

(2) $$\left\{ \begin{array}{l} M_{1}(k)=\frac{V_{IN}(k)-V_{O}(k)}{L}\\ M_{2}(k)=\frac{V_{O}(k)}{L} \end{array}\right.$$

where *M*_1_(*k*) and *M*_2_(*k*) denote the positive slope and the negative slope of the inductor current, respectively. Meanwhile, the rising duration time of inductor current is *D*(*k*)*T*, and the falling duration time is [1−*D*(*k*)]*T*. Thus, the variation of the inductor current in the *k*th switching cycle can be written as Equation (3):

(3) $${\text{Î}}_{OB}(k)=I_{OB}(k+1)-I_{OB}(k)=-M_{2}(k)D'(k)T+M_{1}(k)D(k)T$$

where *I_OB_*(*k*) denotes the observed current, and *D*'(*k*) is an abbreviation for 1−*D*(*k*). Equation (3) is the basic current observer equation, from which the PCC algorithm is also derived.

### 2.2. Predictive Current Control

**Figure 2.**
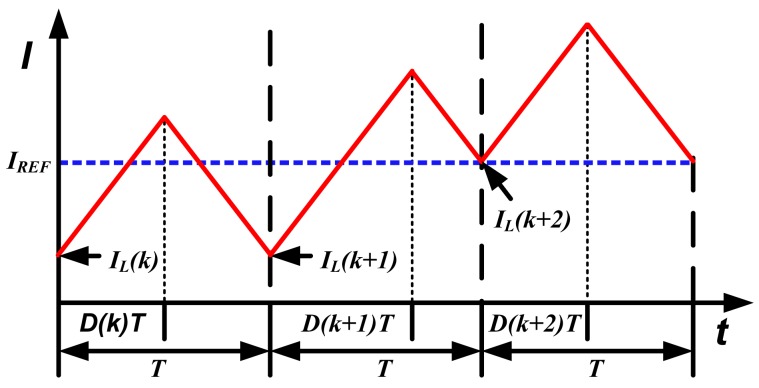
Current waveform under valley current control and TE modulation.

Employing valley current control and trailing edge (TE) modulation, the inductor current waveform is shown in Figure 2.

In Figure 2, *I_REF_* is the reference current output from the PI regulator. There exists a deviation between *I_L_*(*k*) and *I_REF_*. The PCC controller detects the current error, and adjusts the duty ratio *D*(*k*+1), so that *I_L_*(*k*+2) reaches *I_REF_*. In this way, the current error can be eliminated in two switching cycles.

As the switching cycle *T* is much shorter than the regulation time, the inductor current slope can be regarded as constant in two adjacent switching cycles, that is:

(4) $$\left\{ \begin{array}{l} M_{1}(k)=M_{1}(k+1)=M_{1}\\ M_{2}(k)=M_{2}(k+1)=M_{2} \end{array}\right.$$

Substituting the control objective *I_OB_*(*k*+2) = *I_REF_* in Equation (3), we have:

(5) $$D(k+1)=\frac{I_{\text{REF}}-I_{OB}(k+1)+M_{2}T}{(M_{1}+M_{2})T}$$

As shown above, the PCC controller can eliminate the current error in less than two switching cycles, which is relatively fast, so the converter can be designed with fast transient response. However, the CO-based PCC controller has problems such as the divergence of the observed current and the steady state error of the output voltage. In the next section, this issue will be discussed in detail, and an optimal current observation strategy will be given to solve the problems.

## 3. Convergence of the Observed Current and the Steady State Error of the Output Voltage

For the PCC controller with the basic CO, the observed current will diverge due to the diode forward voltage and the parasitic resistors in the converter. The divergence of the observed current degrades the reliability of the converter, and will cause the steady state errors in the output voltage. In the following section, the relationship among the parasitic parameters, the convergence of the observed current and the steady state error of the output voltage will be studied in detail.

### 3.1. Analysis of the Convergence of the Observed Current

The basic CO is based on Equations (2) and (3), thus the variation of the inductor current in one switching cycle can be written as:

(6) $${\text{Î}}_{OB}=I_{OB}(k+1)-I_{OB}(k)=\frac{V_{IN}}{L}DT-\frac{V_{O}}{L}T$$

However, Equation (6) is not precise enough due to the existence of parasitic parameters in the actual circuit. Taking the diode forward voltage *V_F_* into consideration, for example, a more accurate equation for the current variation can be written as:

(7) $${\text{Î}}_{\text{ACT}}=\frac{V_{IN}}{L}DT-\frac{V_{O}}{L}T-\frac{V_{F}}{L}D'(k)T$$

Equation (7) describes the actual current variation of the inductor, which must be zero in the steady state. So, from Equations (6) and (7):

(8) $${\text{Î}}_{OB}=\frac{V_{F}}{L}D'(k)T>0$$

**Figure 3.**
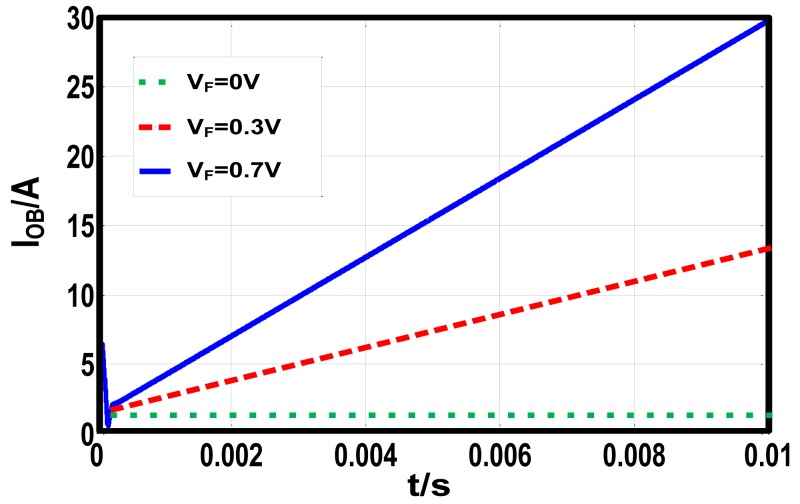
The observed current with different *V_F_*.

Equation (8) indicates that the existence of *V_F_* will cause the divergence of the observed current. The divergence speed depends on *L, D* and *V_F_*. To demonstrate the derivation, a buck DC-DC converter is simulated in Matlab-simulink. The switching cycle is 100 kHz, the input voltage is 10 V, the reference voltage is 6 V and the inductor volume is 100 μH. Employing the basic CO, the observed currents in simulation match Equation (8) precisely, which is shown in Figure 3.

Other parasitic parameters like the inductor winding resistor, the diode forward resistor and the MOS on-resistor will also diverge the observed current. The divergence speed is proportional to those parasitic parameters. The divergence of the observed current further causes the steady state error of the output voltage, which will be analyzed in the following.

### 3.2. Analysis for the Steady State Error of the Output Voltage

To demonstrate the steady state error of the output voltage, the mechanism of PI regulator must be taken into consideration. Adopting Laplacian method, the transfer function of PI controller can be written as (*K_P_sT_I_* + *K_P_*)/*sT_I_*, where *K_P_* and *T_I_* denote the proportional and integral parameters, respectively. The PI regulator accepts the voltage error Δ*V_O_*, and outputs the reference current *I_REF_*, which can be written as:

(9) $$I_{\text{REF}}=\frac{K_{P}sT_{I}+K_{P}}{sT_{I}}\Delta V_{O}$$

In steady state, the output voltage must be constant, which guarantees *s*ΔV_O_ = 0, so the Equation (9) can be written as:

(10) $$I_{\text{REF}}=\frac{K_{P}}{sT_{I}}\Delta V_{O}$$

Rationally, the PI regulator can eliminate the voltage error, and the output *I_REF_* is an integration of zero, which should be a constant value. However, as shown in Section 3.1, the observed current diverges due to the parasitic parameters. In this way, the current error Δ*I* = *I_REF_* − *I_OB_* also diverges, which is not acceptable for the PCC controller. According to Equation (5), the current error Δ*I* must be constant to guarantee a stable duty ratio, thus:

(11) $${\text{Î}}_{OB}=sTI_{OB}=sT(I_{\text{REF}}-\Delta I)=sTI_{\text{REF}}$$

Finally, the steady state error of the output voltage occurs due to the divergence of *I_OB_* can be derived using Equations (10) and (11):

(12) $$\Delta V_{O}=\frac{T_{I}}{K_{P}T}{\text{Î}}_{OB}$$

From Equations (8) and (12), the relation between Δ*V_O_* and *V_F_* can be derived as:

(13) $$\Delta V_{O}=\frac{D'T_{I}}{LK_{P}}V_{F}$$

**Figure 4.**
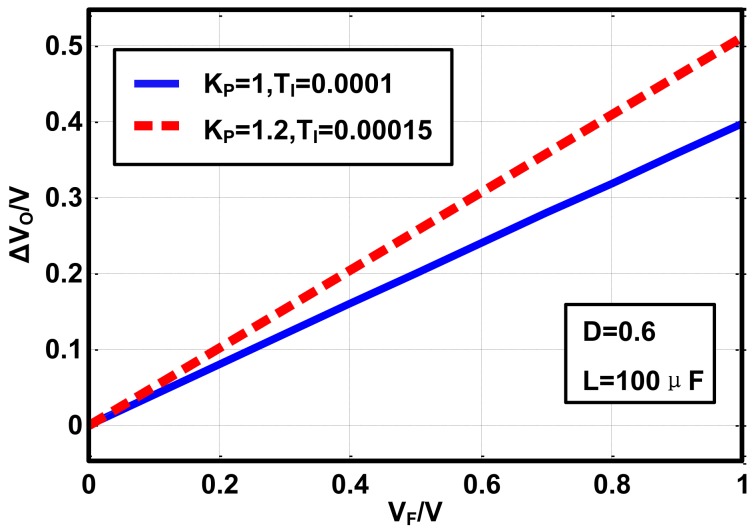
Simulated steady state error of the output voltage under different PI parameters and *V_F_*.

Equations (8) and (13) indicate that both the value of the steady state error and the divergence speed of the observed current are proportional to *V_F_*. To demonstrate this conclusion, a buck converter is constructed under Matlab-simulink with the same specifications as the simulation described in Section 3.1. The current loop employs the basic current observer, while the voltage loop uses the basic PI controller. The steady state error of the output voltage is simulated under different *V_F_* and PI parameters, as is shown in Figure 4.

The slope of the voltage error *versus V_F_* is 0.4 when the PI parameters are set as *K_P_*=1, *T_I_*=0.0001. The slope increases to 0.5 when the PI parameters change to *K_P_*=1.2, *T_I_*=0.00015. The simulated voltage steady state error matches the Equation (13) precisely.

Other parasitic parameters such as the equivalent serial resistor of the inductor, the diode forward resistor, *etc.*, will also increase the steady state error of the output voltage. To solve the problem, and to achieve the highest observation accuracy, the OCO strategy is proposed, and will be introduced in the next section.

## 4. Optimal Current Observer

For the PCC controller with the basic CO, the observed current is found to be diverging, which further induces the steady state error of the output voltage. In this section, the OCO strategy is proposed, which can converge the observed current to the valley value of the inductor current, and eliminate the steady state error of the output voltage.

### 4.1. Compensation for the Observed Current

In order to model the system precisely, several parasitic parameters are considered. Then the current slopes are compensated as:

(14) $$\left\{ \begin{array}{l} M_{1}=\frac{V_{IN}-V_{AV}-I_{AV}(R_{DS}+R_{L})}{L}\\ M_{2}=\frac{V_{AV}+V_{F}+I_{AV}(R_{F}+R_{L})}{L} \end{array}\right.$$

where *V_AV_* and *I_AV_* denote the average values of the output voltage and the inductor current respectively. Equation (14) can be used to calculate the average value of the inductor current. However, the PCC controller works in valley current mode, thus the observed current should be the valley value of the inductor *I_V_*, so taking the inductor current ripple into consideration:

(15) $$\left\{ \begin{array}{l} M_{1}=\frac{V_{IN}-V_{AV}-(I_{V}+I_{pp}/2)(R_{DS}+R_{L})}{L}\\ M_{2}=\frac{V_{AV}+V_{F}+(I_{V}+I_{pp}/2)(R_{F}+R_{L})}{L} \end{array}\right.$$

where the current ripple can be written as *I_pp_*=(1−*D*)*VT*/*L*. Substituting Equation (15) into Equation (3):

(16) $${\text{Î}}_{OB}=I_{OB}(k+1)-I_{OB}(k)=\frac{T}{L}[DV_{IN}-V_{AV}-(I_{OB}+I_{pp}/2)R_{T}-D'V_{F}]$$

where *R_T_*=*R_L_*+*DR_DS_*+*D*'*R_F_* is an equivalent compensation resistance. Based on Equation (16), the OCO shall achieve the highest observation accuracy. More importantly, the observed current no longer diverges, which will be proven next.

Employing first order differential approximation *I_OB_*' ≈ [*I_OB_*(*k*+1) − *I_OB_*(*k*) ]/*T*, Equation (16) can be written in differential equation formula:

(17) LIOB'+RT(IOB+Ipp/2)=DVIN−VAV−D'VF

Therefore, the observed current can be solved as:

(18) $$I_{OB}=\frac{DV_{IN}-V_{O}-D'V_{F}}{R_{T}}-\frac{I_{pp}}{2}+Ae^{-\frac{R_{T}}{L}t}$$

where A is an arbitrary constant. Equation (18) indicates that the observed current necessarily converges to the valley value of the inductor current, that is:

(19) $$I_{OB}{|}_{t\to \infty }=I_{V}=\frac{DV_{IN}-V_{O}-D'V_{F}}{R_{T}}-\frac{I_{pp}}{2}$$

To be strict, the parasitic parameters are always changing with the temperature, the time, *etc.* However, the deviation would not change the convergence of the observed current. The observed current necessarily converges to the value in Equation (19) no matter how the parasitic parameters deviate. Because the derivation above is based on the observer algorithm itself.

### 4.2. Compensation for the Sampled Voltage

It is a natural character of DC-DC converters that the output voltage contains ripples, so the sampled voltage may deviate from the average value of the output voltage, depending on the sampling point. The maximum deviation range is the value of the output voltage ripple. Nevertheless, all the industrial applications take the average value as the reference voltage. What is more, the current observer also prefers *V_AV_* for observation. Thus, the sampled voltage should be compensated to the average voltage. To derive the compensation strategy, the output voltage ripple and the sampling point should be analyzed. For a buck converter, the output voltage ripple (peak-peak value) is [23]:

(20) $$V_{pp}=\frac{I_{pp}T}{8C}$$

where *I_pp_* is the ripple (peak-peak value) of the inductor current. Obviously, the output voltage ripple can be minimized by increasing the output capacitor. Nevertheless, the effect of the capacitor ESR is not considered.

**Figure 5.**
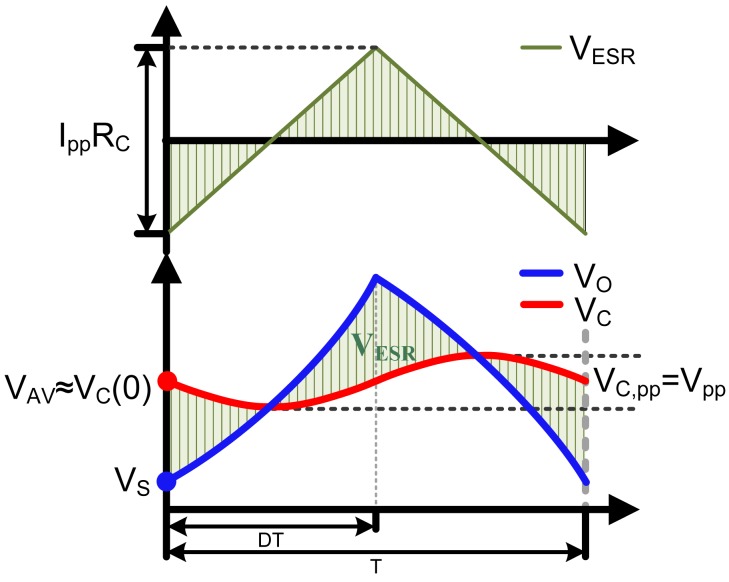
Output voltage and voltage between the ideal capacitor.

As Figure 5 shows, the output voltage *V_O_* is the sum of the voltage on the ideal capacitor *V_C_* and the voltage on the ESR *V_ESR_*, which can be written as:

(21) $$V_{O}(t)=V_{C}(t)+V_{\text{ESR}}(t),(0\leq t\leq T)$$

where *t* denotes the time in one switching cycle. The current that flows through the ESR is the sum of the inductor current and the load current, where the load current can be regarded as constant, so the voltage drop on the ESR can be written as:

(22) $$V_{\text{ESR}}(t)=[I_{L}(t)-\frac{V_{O}(t)}{R}]R_{C}\approx [I_{L}(t)-I_{AV}]R_{C}$$

where *I_AV_* is the average value of the inductor current. The Equations (21) and (22) indicates that, the voltage drop on the ESR affects the output voltage. The output voltage ripple is determined by both the ripple on the capacitor *V_C,pp_* and the ripple on the ESR *V_ESR,pp_*, so it can be written as:

(23) $$\max(V_{C,pp},V_{\text{ESR},pp})\leq V_{O,pp}\leq V_{C,pp}+V_{\text{ESR},pp}$$

In Equation (23), the ripple on the ideal capacitor can be written as *V_C.pp_*=*I_pp_T*/8*C*, which is the same as *V_pp_*. The ripple on the ESR can be derived through the Equation (22), and be *V_ESR,pp_*=*I_pp_R_C_*. So the output voltage ripple *V_O,pp_* is mainly determined by the ESR when the ESR exceeds *T*/8*C*.

The sampled voltage is determined by *V_O_*(*t*) and the sampling point. For the buck converter with TE modulation, the sampling point is conventionally set at the beginning of every switching cycle. Thus the sampled voltage could be written as:

(24) $$V_{S}=V_{O}(0)=V_{C}(0)+V_{\text{ESR}}(0)$$

where *V_S_* denotes the sampled voltage. At the sampling point, *V_C_* is closer to *V_AV_* than *V_S_*. So the compensation strategy can be given as:

(25) $$V_{\text{COMP}}=V_{C}(0)=V_{S}+\frac{1}{2}I_{pp}R_{C}$$

where *I_pp_* can be calculated by the current observer. Taking *V_COMP_* as the voltage average value is relatively acceptable. The error is small, which will be proven next.

To calculate the accurate error between *V_COMP_* and *V_AV_*, expressions for *V_C_*(*t*) and *V_O_*(*t*) must be deduced. Firstly, the inductor current, which is the input signal, could be written as:

(26) $$I_{L}(t)=I_{pp}(\frac{t}{DT}-\frac{1}{2})U(t)+I_{pp}[\frac{(2D-1)t}{DD'T}+\frac{1}{D'}]U(t-DT)$$

Meanwhile, the transfer function from the inductor current to *V_O_* can be written as:

(27) $$H_{vi1}(s)=\frac{R(\frac{1}{sC}+R_{C})}{\frac{1}{sC}+R_{C}+R}=\frac{R(1+sR_{C}C)}{1+sC(R_{C}+R)}$$

And the transfer function from the inductor current to *V_C_* can be written as:

(28) $$H_{vi2}(s)=H_{vi1}(s)\frac{\frac{1}{sC}}{\frac{1}{sC}+R_{C}}=\frac{R}{1+sC(R_{C}+R)}$$

Employing Laplacian method, the expressions for *V_O_*(*t*) and *V_C_*(*t*) can be derived as:

(29) $$\left\{ \begin{array}{l} V_{O}(t)={\text{L}}^{-1}\left(\frac{R(1+sR_{C}C)}{1+sC(R_{C}+R)}\text{L}(I_{L}(t))\right)\\ V_{C}(t)={\text{L}}^{-1}\left(\frac{R}{1+sC(R_{C}+R)}\text{L}(I_{L}(t))\right) \end{array}\right.$$

**Figure 6.**
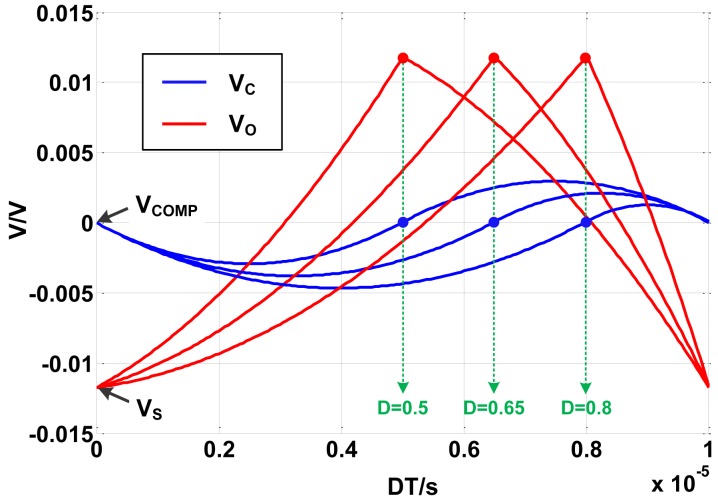
Simulated *V_C_* and *V_O_* using Laplacian method.

**Figure 7.**
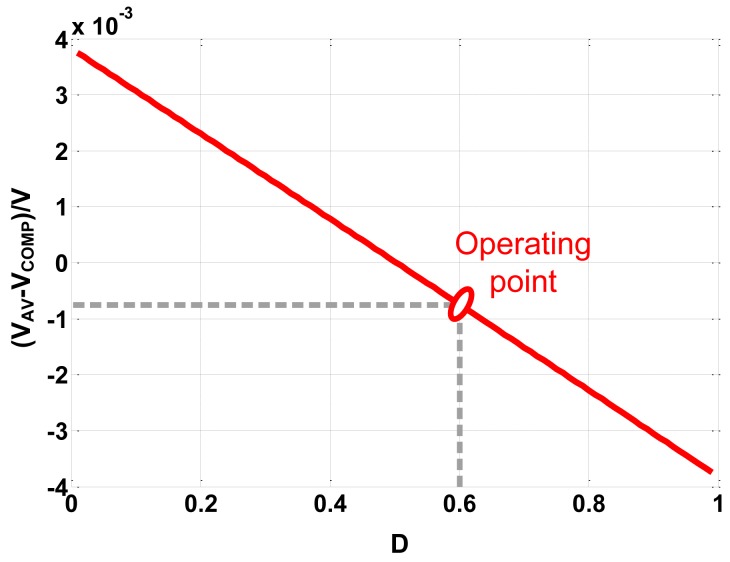
Error between *V_COMP_* and *V_AV_*.

By means of computer assistance analysis, Equation (29) can be used to calculate the accurate value of *V_C_*(*t*) and *V_O_*(*t*). Simulation is carried out based on above derivations. The voltage on the ideal capacitor and the output voltage are plotted in Figure 6. *V_AV_* can be calculated by integrating either *V_C_*(*t*) or *V_O_*(*t*). Then the error between *V_COMP_* and *V_AV_* is plotted under different duty ratios, as Figure 7 shows.

As Figure 6 shows, the error between *V_S_* and *V_COMP_* is about 12 mV, so the error between *V_S_* and *V_AV_* can be written as *V_AV_* − *V_S_* = *V_AV_* – *V_COMP_* + 12 mV. Figure 7 indicates that the absolute value of the error between *V_COMP_* and *V_AV_* is proportional to *D*−0.5. |*V_AV_*−*V_COMP_*| equals to zero when *D* is 0.5, while be maximum value 4 mV when *D* is 0 or 1. At the operating point *D* = 0.6, |*V_AV_*−*V_COMP_*| is less than 1 mV. So the compensated voltage is much closer to the average voltage than the sampled voltage because |*V_AV_*−*V_COMP_*|≪|*V_AV_*−*V_S_*|.

## 5. Experiments

In order to prove the theories about the convergence of the observed current and the steady state error of the output voltage, both the basic CO based PCC controller and the OCO based PCC controller are experimentally demonstrated. Meanwhile, comparison between the OCO based PCC controller and a conventional voltage mode controller is also carried out. In this section, the experimental settings which include the design specifications and the measured parasitic parameters are introduced first. Then the test results are given and analyzed.

The design specifications and the measured parasitic parameters are shown in Tables 1 and 2, respectively. The system hardware includes a control part and a power part. The control part as well as other software features is implemented using the Texas Instruments TMS320F2812 digital signal processor (DSP). The power part includes the main power stage and the signal sampling circuits. The switching device of the power stage is a BSZ110N06NS3 MOSFET, the output capacitor is an EEHZC1E101XP, and the diode is a SB350. The input and output voltages are sampled by a 4 channels 12-bit AD converter chip (AD7934-6). *I_OB_* is output in synchronization with the 12 bit DA chip (TLV5616). The actual inductor current is measured by a Tektronix current probe with a resolution of 1 V/A.

### 5.1. Experiment for the CO Based PCC Controller

**Figure 8.**
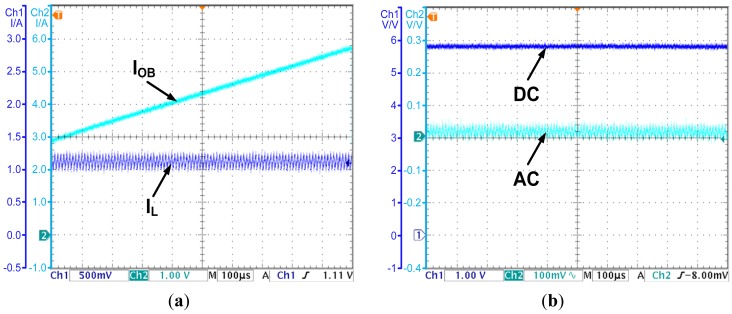
(**a**) The observed current and the inductor current—the CO based PCC controlled converter; (**b**) The output voltage—the CO based PCC controlled converter.

As shown in Section 3, for the basic CO based PCC controlled converter, the observed current will diverge from the actual inductor current due to the parasitic parameters. An experiment is carried out to demonstrate this conclusion. With the CO based PCC controller, the observed current and the actual inductor current are shown in Figure 8a, while the output voltage is shown in Figure 8b.

As Figure 8a shows, the actual inductor current is about 1.2 A. However, the observed current rises with a constant slope due to the parasitic parameters in the actual converter. The divergence of the observed current will cause calculation result overflow in the digital circuit and degrades the reliability of the controller. More importantly, the divergence will cause the steady state error of the output voltage. As Figure 8b shows, the output voltage is 5.8 V, and deviates from the reference voltage 6 V by 0.2 V. The steady state error is 3.3 percent of the output voltage. The results prove the theories proposed in Section 3.

### 5.2. Experiment for the OCO Based PCC Controller

**Figure 9.**
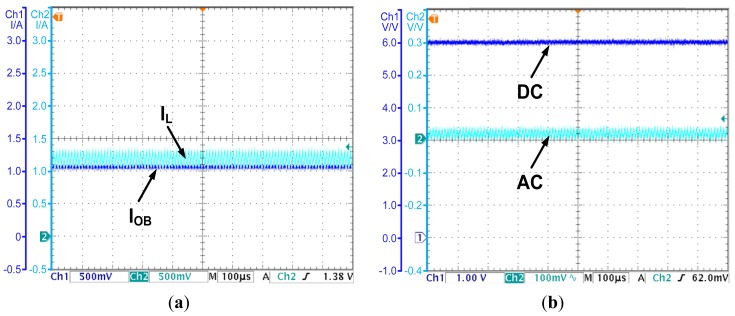
(**a**) The observed current and the inductor current—the OCO based PCC controlled converter; (**b**) The output voltage—the OCO based PCC controlled converter.

The OCO strategy is proposed in Section 4. In the following, an experiment is carried out to demonstrate the effectiveness of the OCO based PCC controller. The observed current is shown in Figure 9a, while the output voltage is shown in Figure 9b.

As Figure 9a shows, by introducing compensations for the parasitic parameters, the observed current converges to the valley value of the inductor current. The error is less than 0.05 A. The accuracy is acceptable for applications. As Figure 9b shows, the OCO based PCC controller regulates the average value of the output voltage to the reference voltage 6 V. The steady state error is eliminated. All the results prove the effectiveness of OCO and the theories proposed in Section 4.

### 5.3. Experiment of System Robustness

**Figure 10.**
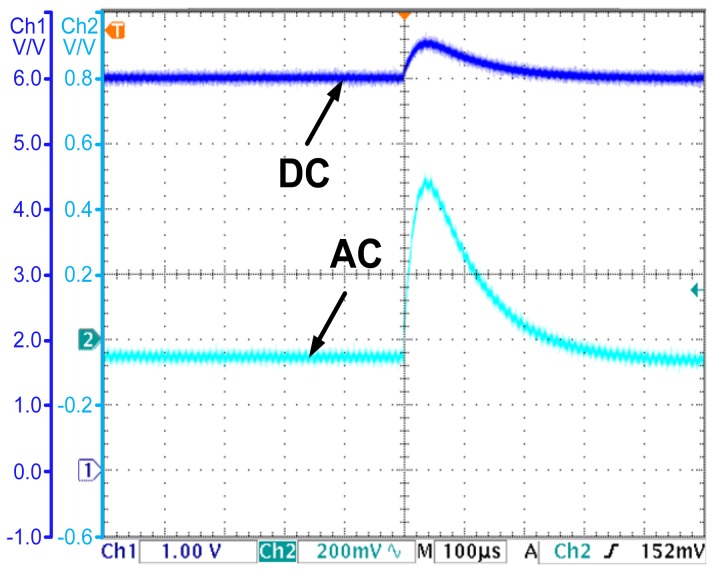
The output voltage transient when the load steps from 3 to 5 Ω—a conventional voltage mode controlled converter.

**Figure 11.**
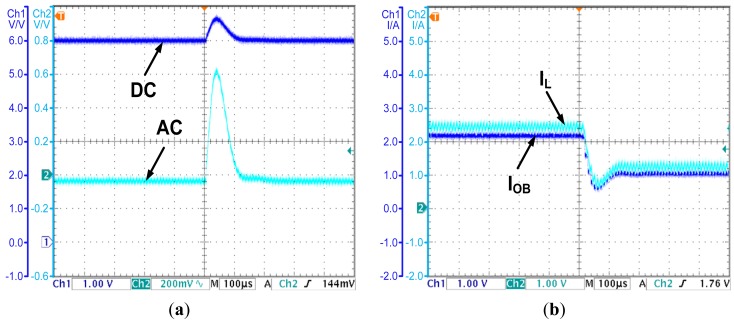
(**a**) The output voltage transient when the load steps from 3 Ω to 5 Ω—the OCO based PCC controlled converter; (**b**) The observed current and the inductor current transient when the load steps from 3 to 5 Ω—the OCO based PCC controlled converter.

To demonstrate the advantage of the OCO-based PCC controller in dynamic response speed, a comparison is made between the proposed controller and a conventional voltage mode controller. The test results are as follows: when the load steps from 3 to 5 Ω, the output voltage of the conventional voltage mode controlled converter increases to 6.55 V for a short time, and re-stabilizes in 400 μs, which is shown in Figure 10. For the OCO-based PCC controlled converter, the output voltage and the inductor current under the same disturbance are shown in Figure 11a,b, respectively. The output voltage increases to 6.7 V for a short time, and re-stabilizes in 200 μs, while the inductor current reaches 1.2 A in less than 150 μs. Compared to the conventional voltage mode controller, the OCO- based PCC controller can cut the regulation time by 200 μs.

**Figure 12.**
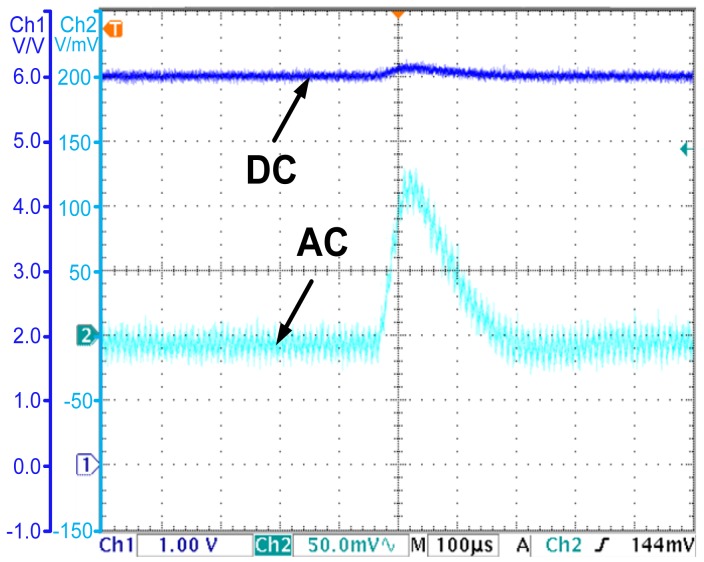
The output voltage transient when the input voltage steps from 10 to 12 V—a conventional voltage mode controlled converter.

**Figure 13.**
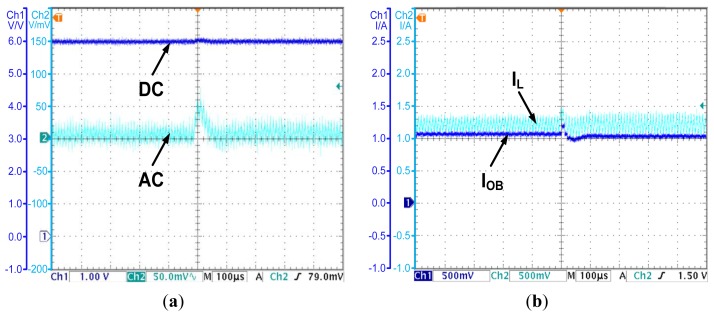
(**a**) The output voltage transient when the input voltage steps from 10 to 12 V—the OCO based PCC controlled converter; (**b**) The observed current and the inductor current transient when the input voltage steps from 10 to 12 V—the OCO based PCC controlled converter.

The output voltage transient of the conventional voltage controlled converter when the input voltage steps from 10 to 12 V is shown in Figure 12. The output voltage increases to 6.12 V for a short time, and re-stabilizes in 250 μs. Finally, for the OCO-based PCC controlled converter, the output voltage and the inductor current under the same disturbance are shown in Figure 13a,b, respectively. The output voltage increases to 6.05 V for a short time, and re-stabilizes in 100 μs, while the inductor current re-stabilizes in 100 μs. Compared to the conventional voltage mode controller, the OCO based PCC controller can cut the regulation time by 150 μs.

As the experimental results show, the proposed strategy can improve the transient response of the buck DC-DC converter.

## 6. Conclusions

A current observer is a possible substitute for conventional current sensors in digitally controlled DC-DC converters. This paper studied the divergence problem of the observed current based on the PCC controlled buck DC-DC converter. Meanwhile, the divergence-induced voltage steady state error is analyzed. In order to solve these issues, the OCO strategy is proposed. It achieves the highest observation accuracy based on compensations for all the known parasitic parameters. Experimental results demonstrate that by employing the OCO-based PCC controller, the observed current converges to the valley value of the inductor current, and the steady state error of the output voltage is also eliminated. Moreover, compared to the conventional voltage mode controller, the proposed algorithm can improve the transient response, which is proven to have a good theoretical and practical application potential for CCM buck converters.

## Figures and Tables

**Table 1.** Design specifications of the buck converter.

Input voltage	10 V
Output voltage	6 V
Rated output current (ROC)	1.2 A
Voltage ripple under ROC	0.12%
Switching frequency	100 kHz
Inductance of the power inductor	100 μH
Capacitance of the output capacitor	50 μF

**Table 2.** Measured parasitic parameters.

Inductor winding resistance *R_L_*	200 mΩ
MOSFET *R_DS_*	100 mΩ
Diode forward resistance *R_F_*	100 mΩ
Diode forward Voltage *V_F_*	0.7 V
ESR value of the output capacitor *R_C_*	70 mΩ
